# PCR primers for 30 novel gene regions in the nuclear genomes of Lepidoptera

**DOI:** 10.3897/zookeys.596.8399

**Published:** 2016-06-08

**Authors:** Niklas Wahlberg, Carlos Peña, Milla Ahola, Christopher W. Wheat, Jadranka Rota

**Affiliations:** 1 Department of Biology, University of Turku, 20014 Turku, Finland; 2 Department of Biology, Lund University, 223 62 Lund, Sweden; 3 Department of Zoology, Stockholm University, 106 91 Stockholm, Sweden

**Keywords:** Molecular systematics, Lepidoptera, phylogenomics, phylogenetics

## Abstract

We report primer pairs for 30 new gene regions in the nuclear genomes of Lepidoptera that can be amplified using a standard PCR protocol. The new primers were tested across diverse Lepidoptera, including nonditrysians and a wide selection of ditrysians. These new gene regions give a total of 11,043 bp of DNA sequence data and they show similar variability to traditionally used nuclear gene regions in studies of Lepidoptera. We feel that a PCR-based approach still has its place in molecular systematic studies of Lepidoptera, particularly at the intrafamilial level, and our new set of primers now provides a route to generating phylogenomic datasets using traditional methods.

## Introduction

Post-Sanger sequencing technologies have opened up vast possibilities for acquiring molecular data for inferring phylogenetic relationships among taxa using 100s to 1000s of loci (Lemmon and Lemmon 2013), from whole genome sequences (e.g. Jarvis et al. 2014), to whole transcriptome sequences (e.g. Misof et al. 2014), to the targeted capture of conserved regions in genomes (e.g. Prum et al. 2015). However, these approaches require high quality DNA or RNA extracted from samples that are fresh or have been stored appropriately. Unfortunately, this quality requirement fails to capitalize on the wealth of material collected and cataloged in museums around the world. Recognizing this, many researchers are currently attempting to develop protocols to allow the extraction and sequencing of large amounts of DNA from old museum samples (e.g. Timmermans et al. 2016), but these methods are primarily limited to mitochondrial DNA.

For the past two decades, the standard protocol in insect molecular systematics has been to extract genomic DNA from one or two legs of dried individuals, often several years old, generally yielding very low concentrations of DNA. Today, millions of such genomic DNA extracts exist, each taken from suboptimally stored specimens, generated by individual researchers and large facilities such as the Canadian Centre for DNA Barcoding. These extracts have been used to PCR amplify specific gene regions, followed by Sanger sequencing. This standard approach has traditionally been restricted to fewer than 10 gene regions due to the lack of universal primers for more regions. Given this extensive DNA resource and the inability of the aforementioned methods to be easily applied to them, here we present an approach for using these extracts in the pursuit of phylogenomic insights.

As DNA sequencing technologies continue to evolve, the molecular systematist must judiciously choose which tools are best suited to the questions they wish to address. While genome scale data are certainly useful, such data are expensive, difficult to analyze and ultimately only a small fraction is utilized. Perhaps most importantly, such large scale datasets are likely only necessary for resolving deeper evolutionary events, such as the relationships among orders of insects (Misof et al. 2014) or superfamilies of e.g. Lepidoptera (Bazinet et al. 2013; Kawahara and Breinholt 2014). In contrast, datasets on the order of tens of genes have been highly useful for resolving relationships at the intrafamilial level, as has been repeatedly shown for e.g. lepidopteran families (Wahlberg et al. 2009, 2014; Kaila et al. 2011; Kawahara et al. 2011; Sihvonen et al. 2011; Zahiri et al. 2011, 2012; Zwick et al. 2011; Regier et al. 2012a, 2012b; Rota and Wahlberg 2012; Sohn et al. 2013). Thus, datasets generated with PCR-based methods have been and continue to be very insightful. However, in many such studies, some nodes are poorly supported with the scale of data available and more sequence data is needed. But, while it would be very interesting to sequence e.g. transcriptomes for the same species sampled, financial and practical constraints preclude such attempts. Rather, what is most likely to help resolve many of these ambiguities in a cost effective and timely fashion are more high quality loci that can be amplified with PCR across a range of DNA quality.

Whole genome sequences can now be used to search for suitable gene regions for primer design (e.g. Wahlberg and Wheat 2008). Such suitable gene regions are considered to be protein-coding genes that are single copy and have an exon that is longer than 500 bp. Long exons are needed as intron lengths can vary thousand fold between taxa, sometimes even between close relatives (Zhang and Hewitt 2003). Protein-coding genes are also preferred for inferring phylogenetic relationships as their alignments are generally unambiguous and conserved regions can be found for primer design.

Here we design and test PCR primers for long exon regions of single copy, protein-coding genes across Lepidoptera based on publicly available whole genome sequences of the order. The new gene regions are shown to be phylogenetically informative for Lepidoptera and can be used to complement the eight gene regions that have become standard in Lepidoptera phylogenetics (Wahlberg and Wheat 2008).

## Material and methods

Single copy, protein-coding genes with exons longer than 500 bp were found while manually curating the set of genes listed in Misof et al. (2014) that were pulled out of eight publicly available Lepidoptera genomes using *tblastn*: *Bombyx mori* (NCBI accession GCA_000151625), *Plutella xylostella* (GCA_000330985), *Manduca sexta* (GCA_000262585), *Danaus plexippus* (GCA_000235995), *Heliconius melpomene* (GCA_000313835), *Melitaea cinxia* (GCA_000716385), *Chilo suppressalis* (GCA_000636095), and *Spodoptera frugiperda* (GCA_000753635). Sequences from all eight genomes were then used to design universal primers. We used the Python library primer-designer v0.2.0 (Peña 2015) to submit batches of FASTA alignments to the website primer4clades (Contreras-Moreira et al. 2009) in order to retrieve candidate primers for each gene sequence. Primer selection was based on high quality and amplicon length between 200 and 500 bp.

As in Wahlberg and Wheat (2008), universal tails were added to all primers to facilitate sequencing. Primers were aliquoted to a standard concentration of 10 μM for use. Primers were tested on a set of 24 species of Lepidoptera that represent major lineages within the order (Table 1). The DNA extracts of these specimens were previously used in the study by Mutanen et al. (2010). They mainly come from small amounts of tissue (such as legs) preserved in 100% EtOH (details of preservation and extraction methods can be found in Mutanen et al. 2010). The PCR reactions for all samples were done using the MyTaq^TM^ HS Red Mix (Bioline) in a final volume of 12.5 µl per sample. For each reaction we used 4 µl of MQ-H_2_O, 6.25 µl of 2x MyTaq HS Red Mix, 0.625 µl of both forward and reverse primers and 1 µl of extracted DNA. All primers were tested with a standard thermal cycling profile of 95 °C for 7 minutes, then 40 cycles of 94 °C for 30 seconds, 55 °C for 30 seconds, 72 °C for 2 minutes, with a final extension period of 72 °C for 10 minutes. A standard cycling profile was chosen to simplify procedures and allow for large scale testing. No optimization of PCR reactions was attempted, as the goal was to find primers that work under exactly the same conditions, allowing the efficient processing of large numbers of samples in the laboratory without having to keep track of specific protocols for specific primer pairs. Success of PCR was visualized on agarose gels and successful PCR products were cleaned enzymatically and sent to Macrogen Europe (Amsterdam) for Sanger sequencing.

**Table 1.** Taxa used to test the primers for amplifying the new gene regions. The last column summarizes the number of new gene regions sequenced for each specimen. See Suppl. material 1 for information about which gene regions were successful.

Voucher code	Family	Genus	Species	Number of new genes sequenced
**MM00058**	Micropterigidae	*Micropterix*	*aureatella*	11
**MM00867**	Nepticulidae	*Ectoedemia*	*occultella*	18
**MM00943**	Tischeriidae	*Tischeria*	*ekebladella*	18
**MM02175**	Psychidae	*Taleporia*	*tubulosa*	22
**MM00030**	Gracillariidae	*Gracillaria*	*syringella*	26
**MM00306**	Yponomeutidae	*Yponomeuta*	*evonymellus*	27
**MM00510**	Tortricidae	*Tortrix*	*viridana*	22
**MM00014**	Schreckensteiniidae	*Schreckensteinia*	*festaliella*	26
**MM02524**	Epermeniidae	*Epermenia*	*illigerella*	24
**MM03096**	Pterophoridae	*Stenoptilia*	*veronicae*	22
**MM00913**	Alucitidae	*Alucita*	*hexadactyla*	19
**MM03941**	Choreutidae	*Choreutis*	*pariana*	21
**MM00021**	Urodidae	*Wockia*	*asperipunctella*	17
**MM00116**	Cossidae	*Cossus*	*cossus*	28
**MM00125**	Sesiidae	*Synanthedon*	*scoliaeformis*	29
**MM00312**	Zygaenidae	*Adscita*	*statices*	26
**MM00034**	Hesperiidae	*Pyrgus*	*malvae*	24
**MM00042**	Elachistidae	*Ethmia*	*pusiella*	25
**MM00051**	Pyralidae	*Pyralis*	*farinalis*	24
**MM00027**	Drepanidae	*Thyatira*	*batis*	28
**MM00032**	Geometridae	*Cyclophora*	*punctaria*	26
**MM00394**	Endromidae	*Endromis*	*versicolora*	29
**MM01170**	Noctuidae	*Apamea*	*crenata*	27
**MM02696**	Lasiocampidae	*Poecilocampa*	*populi*	24

Sequences were trimmed of primer sequences and aligned by eye with reference to amino acid sequence in BioEdit 7 (Hall 1999) using the sequence from *Bombyx mori* as a reference for each gene. Aligned sequences were stored and curated using VoSeq (Peña and Malm 2012).

## Results

We selected a total of 48 gene regions (see Supplementary material for alignments) for primer design, of which 30 successfully amplified (Suppl. material 1 and 2) with the designed primers (Table 3). Only two gene regions were successfully amplified from all 24 test samples (ArgKin and DDX23), but the majority were successfully amplified from 20 or more samples (Table 2). The least successful gene region was LeuZip, which was sequenced from only 9 samples. No samples amplified all 30 gene regions (Table 1); the average number of successful gene regions was about 23. The least successful sample was *Micropterix* (11 out of 30 gene regions sequenced), which is not surprising as the primer design was based on ditrysian species, while *Micropterix* is likely to be the sister group to all the rest of Lepidoptera (Kristensen et al. 2015; Regier et al. 2015). The new gene regions give a total of 11,043 bp of data. The average amplicon length is 368 bp (ranging from 178 to 729 bp).

**Table 2.** Basic information about the new gene regions amplified and sequenced in this study, along with the traditional eight genes used in many previous studies for comparison.

Gene name	Length (bp)	Number of specimens successful	Variable (%)	Pars. Inf. (%)	Conserved (%)	Freq. A (%)	Freq. T (%)	Freq. C (%)	Freq. G (%)	GeneID from *Bombyx* genome
**AFG3a**	336	22	39.3	37.5	60.7	28.0	27.7	20.2	24.1	BGIBMGA010088
**AFG3b**	300	11	47.3	39.7	52.7	34.9	20.9	20.7	23.6	BGIBMGA010088
**ANK13C**	330	20	49.1	38.8	50.9	33.0	28.5	16.4	22.2	BGIBMGA007536
**ArgK**	388	24	44.6	33.5	55.4	22.9	19.0	32.1	26.1	BGIBMGA005812
**Ca-ATPase**	444	23	37.2	30.2	62.8	24.9	21.0	30.1	24.0	BGIBMGA000408
**Ca2**	410	18	44.9	38.5	55.1	33.2	23.6	18.5	24.7	BGIBMGA006603
**chitinase**	405	18	47.2	40.5	52.8	25.7	27.4	23.8	23.2	BGIBMGA008709
**Cullin5**	327	22	48.3	41.0	51.7	33.4	28.7	17.5	20.5	BGIBMGA011511
**CycY**	375	18	39.7	35.5	60.3	29.9	31.4	17.2	21.6	BGIBMGA005969
**DDX23**	303	24	46.9	43.2	53.1	40.4	22.6	13.8	23.2	BGIBMGA003429
**Exp1**	729	15	43.6	35.8	56.4	31.4	28.2	19.5	21.0	BGIBMGA010657
**FCF1**	173	17	49.7	42.8	50.3	32.4	27.7	16.2	23.7	BGIBMGA010318
**GLYP**	384	14	52.3	44.0	47.7	27.2	24.8	25.1	22.9	BGIBMGA010361
**KRR1**	283	16	47.0	39.2	53.0	35.4	26.4	18.0	20.2	BGIBMGA005381
**LeuZip**	372	9	49.5	35.8	50.5	36.4	24.8	18.0	20.9	BGIBMGA003300
**MK6**	255	20	52.2	45.1	47.8	32.8	28.1	18.6	20.6	BGIBMGA005641
**MMP41**	285	21	56.5	48.1	43.5	31.1	30.6	19.7	18.6	BGIBMGA007574
**MPP2**	330	21	44.9	40.3	55.2	29.0	29.4	22.6	19.1	BGIBMGA008312
**NC**	573	15	48.9	39.6	51.1	32.2	29.1	17.0	21.7	BGIBMGA005035
**Nex9**	420	21	60.5	47.4	39.5	33.1	24.8	19.0	23.2	BGIBMGA001032
**PolII**	360	22	43.9	39.4	56.1	30.1	25.3	19.7	24.8	BGIBMGA004994
**ProSup**	432	22	58.8	47.5	41.2	25.6	27.8	21.0	25.6	BGIBMGA004645
**PSb**	366	23	54.4	45.9	45.6	24.8	23.9	26.7	24.7	BGIBMGA000201
**SARAH**	381	16	56.4	44.9	43.6	29.2	27.8	23.3	19.7	BGIBMGA011095
**Ssu72**	249	23	55.0	48.2	45.0	36.0	28.1	16.0	19.9	BGIBMGA000925
**TIF3Cb**	324	13	50.6	40.1	49.4	24.7	22.1	28.9	24.3	BGIBMGA012851
**TIF6**	336	18	50.0	42.6	50.0	24.4	21.4	25.5	28.8	BGIBMGA009830
**UDPG6DH**	405	21	49.1	41.0	50.9	30.1	27.4	20.9	21.6	BGIBMGA012188
**VPS4**	432	15	40.7	35.4	59.3	28.9	28.9	20.1	22.1	BGIBMGA005930
**WD40**	339	21	42.5	38.6	57.5	30.1	31.4	19.3	19.2	BGIBMGA006243
**Genes from Wahlberg and Wheat (2008) for comparison**										
**CAD**	826	24	52.4	42.7	47.6	35.9	28.3	14.6	21.2	
**COI**	1476	23	44.4	33.0	55.6	31.1	40.0	14.9	14.0	
**EF1a**	1047	21	34.9	27.2	65.1	25.4	23.0	27.6	24.0	
**GAPDH**	691	12	38.9	30.8	61.1	23.6	25.8	27.3	23.3	
**IDH**	722	23	48.2	41.1	51.8	31.2	27.1	19.8	21.9	
**MDH**	407	23	47.9	41.3	52.1	27.4	25.8	22.7	24.1	
**RpS5**	603	20	38.5	34.3	61.5	25.4	24.9	24.4	25.3	
**wingless**	400	20	58.5	48.5	41.5	21.7	18.3	28.9	31.0	

**Table 3.** Primers for 30 new gene regions with universal tails (T7promoter-TAATACGACTCACTATAGGG to forward primers and T3-ATTAACCCTCACTAAAGGG to reverse primers) attached to the 5’ end. F = Forward, R = Reverse. Gene names from Table 2.

Gene	Primer
**AFG3a_F**	TAATACGACTCACTATAGGGTGTGAAGAAGCTAAGatwgaratyatggartt
**AFG3a_R**	ATTAACCCTCACTAAAGGGTGTTGTTGTATTAAAAccrtccatytchac
**AFG3b_F**	TAATACGACTCACTATAGGGTGCTCAAGACGACCtdaaraaratmac
**AFG3b_R**	ATTAACCCTCACTAAAGGGCCTGTACCTTCCACGaaytcytcrtamgt
**ANK13C_F**	TAATACGACTCACTATAGGGCAAATACAAAATTTTTATATGGAAytdaartgggaytt
**ANK13C_R**	ATTAACCCTCACTAAAGGGGCAACTGTTTCTTTTCTAtcytcwcgraadatcca
**ArgK_F**	TAATACGACTCACTATAGGGyGAyCCsATCATyGAGGACTACCA
**ArgK_R**	ATTAACCCTCACTAAAGGGAGrTGGTCCTCCTCrTTGCACCAvAC
**Ca2_F**	TAATACGACTCACTATAGGGAAACAGTGGACtgyttgaaraarttcaayg
**Ca2_R**	ATTAACCCTCACTAAAGGGGGTGTGTTGTCGATGaaraayttrtgraa
**Ca-ATPase_F**	TAATACGACTCACTATAGGGGAAtacgarccbgaaatgggwaargt
**Ca-ATPase_R**	ATTAACCCTCACTAAAGGGcdccrtgrgcggggtcgttraagtg
**chitinase_F**	TAATACGACTCACTATAGGGGGTGGGTGCTtayttygtngaatgggg
**chitinase_R**	ATTAACCCTCACTAAAGGGTGTCCACAccrtcraaraayttcca
**Cullin5_F**	TAATACGACTCACTATAGGGTGTTAGTTAAAGATGCTTTTATGgaygaycchmg
**Cullin5_R**	ATTAACCCTCACTAAAGGGTCTTAACCATTCAaccatrtcytcttcyttytc
**CycY_F**	TAATACGACTCACTATAGGGgattatgayaartataatccwgaacayaaaca
**CycY_R**	ATTAACCCTCACTAAAGGGcattgcytcyaatttytgtgcyctttcytt
**DDX23_F**	TAATACGACTCACTATAGGGACAAAAGATAAAGAACGTgargargargchat
**DDX23_R**	ATTAACCCTCACTAAAGGGTGATCTTTTTCAgaccartghckrtcatccca
**Exp1_F**	TAATACGACTCACTATAGGGgthaataaaytdtttgaattyatgcatga
**Exp1_R**	ATTAACCCTCACTAAAGGGggrtaytcttcaaartctttrttdatcat
**FCF1_F**	TAATACGACTCACTATAGGGACTGGACATCGtdcarartatgatggayt
**FCF1_R**	ATTAACCCTCACTAAAGGGTTGTAGCCACGATGtarcayttrtgytg
**GLYP_F**	TAATACGACTCACTATAGGGACTGCGACAAGAAtayttyatgtgygcbgc
**GLYP_R**	ATTAACCCTCACTAAAGGGTTCACTCGTTTTTCACCTtcytcytcdat
**KRR1_F**	TAATACGACTCACTATAGGGaatgcktggrctatgaaratwcc
**KRR1_R**	ATTAACCCTCACTAAAGGGtdataatrtcrcatccwatttcrtc
**LeuZip_F**	TAATACGACTCACTATAGGGTGCCTGTCACAAaaygaytggaaryt
**LeuZip_R**	ATTAACCCTCACTAAAGGGTTTGACCAGGGTTTttdgcrtarttraa
**MK6_F**	TAATACGACTCACTATAGGGTTAGAGAAGGTGATgtntggathtgyatgga
**MK6_R**	ATTAACCCTCACTAAAGGGTTCTTTCTGGTGCCATGtanggyttrca
**MMP41_F**	TAATACGACTCACTATAGGGGAAAACTGGGGTGCTAAagtdtayttyaaya
**MMP41_R**	ATTAACCCTCACTAAAGGGTCACTTTGtttttrttytchccaaawgtcat
**MPP2_F**	TAATACGACTCACTATAGGGCACTTCCGAATCccdtggttycartaycc
**MPP2_R**	ATTAACCCTCACTAAAGGGCCACAGCAGCTGTGtaytcyttdccraa
**NC_F**	TAATACGACTCACTATAGGGgatgaagaaaaycchaaraarttytt
**NC_R**	ATTAACCCTCACTAAAGGGacwatdgaccartggaarttcatdgc
**Nex9_F**	TAATACGACTCACTATAGGGTGCAACTGCAAgartttgtngaytggatg
**Nex9_R**	ATTAACCCTCACTAAAGGGCCCAGTCGTATTTAggytgbtcntcatacat
**PolII_F**	TAATACGACTCACTATAGGGCTGAAACACCTACAatggcbathgaytgggt
**PolII_R**	ATTAACCCTCACTAAAGGGGCTGTAGGGTTCCATttdgcrtgytcytt
**ProSup_F**	TAATACGACTCACTATAGGGGACAACAATCGACtggcayccnaayaa
**ProSup_R**	ATTAACCCTCACTAAAGGGCTGTCCAGTgactggaayttyttcatdgc
**PSb_F**	TAATACGACTCACTATAGGGGCTGGGAGCTACTggvtgytggtgygaya
**PSb_R**	ATTAACCCTCACTAAAGGGAGATGCAGTCTCCAGTGTAGatrtcdckytc
**SARAH_F**	TAATACGACTCACTATAGGGGAAGATGGTATGCCTAATAtwcaycchaayat
**SARAH_R**	ATTAACCCTCACTAAAGGGGTTCACCTTCTTCACGAggytcccadccna
**Ssu72_F**	TAATACGACTCACTATAGGGCAGCTGACAGACCTaaytgttaygarttygg
**Ssu72_R**	ATTAACCCTCACTAAAGGGCCGATTGTAGCTTCTtcrtgrttrtcytg
**TIF3Cb_F**	TAATACGACTCACTATAGGGGAAAAATCGACCACCTGtaytayaarttyga
**TIF3Cb_R**	ATTAACCCTCACTAAAGGGGCCAGCAGTTCTTTAggyttnccvgtcatca
**TIF6_F**	TAATACGACTCACTATAGGGCTGTGCGAGTGcarttygaraayaataa
**TIF6_R**	ATTAACCCTCACTAAAGGGTGTGTCAGCCAGGatytcytchgtrtc
**UDPG6DH_F**	TAATACGACTCACTATAGGGCAGGAACTGTGTtgggtvtaygarcaytg
**UDPG6DH_R**	ATTAACCCTCACTAAAGGGTCTTGTGTCGCCTgtrttyttyttraa
**WD40_F**	TAATACGACTCACTATAGGGGATCCACTTCACAcaygcyaaraayac
**WD40_R**	ATTAACCCTCACTAAAGGGCCTgtccartcacaytcyttytcttg
**VPS4_F**	TAATACGACTCACTATAGGGTGATTCTGATGATCCAGAAaaraaraaryt
**VPS4_R**	ATTAACCCTCACTAAAGGGCATCCATATCAttvccdacaccttgcatytg

The variability in the new gene regions appears to be similar to the widely used nuclear gene regions reported in Wahlberg and Wheat (2008) (Table 2). The base content across most of the fragments is fairly even, with some of them having a small AT bias (e.g., Exp1, CycY, and TIF3Cb), but none having a larger percentage than for example CAD, one of the widely used gene regions (Wahlberg and Wheat 2008), which has 64.1% of As and Ts. The number of parsimony informative sites ranges from 30.2% in Ca-ATPase to a little over 48% in MMP41 and Ssu72. For comparison, the range of parsimony informative sites in the Wahlberg and Wheat (2008) gene regions is almost the same, from 27.2% (EF1alpha) to 48.5% (wingless).

## Discussion

We report here primers for 30 new nuclear gene regions that can be used to complement existing molecular data for Lepidoptera systematics. Our primers were designed to amplify gene regions across the entire taxonomic array of Lepidoptera and to work on relatively degraded material by amplifying less than 500 bp segments of the genome. Many of these primers are being used successfully in our laboratory for projects on e.g. the nymphalid subfamily Limenitidinae (Dhungel and Wahlberg in prep.), the families Geometridae (Brehm et al. in prep.), Choreutidae (Rota et al. in prep.), Limacodidae (Dupont et al. in prep.) and Riodinidae (Seraphim et al. in prep.). The phylogenetic utility of the used gene regions will be reported in more detail in the forthcoming papers: in summary, they are providing similar resolution as the standard gene regions reported in Wahlberg and Wheat (2008) in preliminary maximum likelihood analyses with RAxML that have been conducted.

We would like to stress that the gene regions described here should be seen as complementary to the standard gene regions (Wahlberg and Wheat 2008) and could be used in the event that more data is needed. Potential users should consult Suppl. material 1 to see which primers worked for taxa they are interested in. Based on our experiences in the laboratory, we would recommend that researchers consider using primers for AFG3a, ArgKin, Ca-ATPase, DDX23, MMP41, MPP2, Nex9, PolII, ProSup, PSb, SSU72, UDPG6DH, and WD40, as these tend to amplify consistently, especially across Ditrysia.

More specifically, it seems that several fragments are not very suitable for nonditrysians (none of the three exemplars that we used amplified AFG3b, CHITINASE, KRR1, NC, SARAH, VPS4) and the utility of several other fragments for these groups needs to be further tested (Ca2, GLYP, MPP2, NEX9, POLII, TIF3CB, and UDPG6DH amplified in only one of the nonditrysians tested). On the other hand, 21 fragments amplified in four or more of the six exemplars of Macroheterocera (the exceptions being LeuZip, which amplified in only one of them, and TIF3Cb and VPS4, which amplified in three out of six). The situation is more complex across the lower ditrysians and apoditrysians, which can be expected since these groups are quite divergent (Mutanen et al. 2010; Regier et al. 2013). For these groups our recommendation is to try CHITINASE and MK6 in addition to the above-mentioned fragments that appear to work across Lepidoptera.

In this study, we have used traditional Sanger sequencing to acquire the DNA sequences. However, almost all of the amplicons are short enough to be multiplexed and sequenced on a NextGen sequencing platform, such as Illumina. The advantages would be quick generation of a large number of sequences for a large number of samples. On the other hand, many systematists do not have access to NextGen sequencers, or the bioinformatics knowhow to process the raw data into useable formats, in which case the traditional PCR-based Sanger sequencing approach is still appropriate.

The approach we have used is highly conservative, as we sought to find primer pairs that work under standard conditions. It would thus be possible to design primers for the 18 gene regions that did not work under our strict criteria, but would work under different conditions. It is also possible to design primers that would amplify a longer segment of DNA, although such primer pairs would require fresh samples with little degradation of genomic DNA. It would also be possible to find more gene regions with exon lengths more than 500 bp, although a PCR-based approach becomes less and less efficient as the number of reactions grows. It is quite likely that datasets comprising up to 20 gene regions are sufficient for most phylogenetic studies within families (Zwick et al. 2011). For more difficult phylogenetic problems, NextGen sequencing approaches are recommended.

## Supplementary materials

Supplementary material 1 Table S1 Data type: NCBI accession numbers Explanation note: Details of the success of sequencing of the new gene regions. GenBank accession number indicates successful sequencing, dash indicates unsuccessful amplification. This dataset is made available under the Open Database License (http://opendatacommons.org/licenses/odbl/1.0/). The Open Database License (ODbL) is a license agreement intended to allow users to freely share, modify, and use this Dataset while maintaining this same freedom for others, provided that the original source and author(s) are credited.Niklas Wahlberg, Carlos Peña, Milla Ahola, Christopher W. Wheat, Jadranka Rota

Supplementary material 2 Sequences used for designing primers Data type: Reference sequences Explanation note: A zip-file containing reference sequences for all 48 gene regions used for designing primers. This dataset is made available under the Open Database License (http://opendatacommons.org/licenses/odbl/1.0/). The Open Database License (ODbL) is a license agreement intended to allow users to freely share, modify, and use this Dataset while maintaining this same freedom for others, provided that the original source and author(s) are credited.Niklas Wahlberg, Carlos Peña, Milla Ahola, Christopher W. Wheat, Jadranka Rota
